# Safety, Immunogenicity, and Effectiveness of Defective Viral Particles Arising in Mast Cells Against Influenza in Mice

**DOI:** 10.3389/fimmu.2020.585254

**Published:** 2020-11-13

**Authors:** Caiyun Huo, Jijing Tian, Jinlong Cheng, Jin Xiao, Mingyong Chen, Shumei Zou, Haiyan Tian, Ming Wang, Huiling Sun, Yanxin Hu

**Affiliations:** ^1^ Key Laboratory of Animal Epidemiology of Ministry of Agriculture, College of Veterinary Medicine, China Agricultural University, Beijing, China; ^2^ Beijing Key Laboratory for Prevention and Control of Infectious Diseases in Livestock and Poultry, Institute of Animal Husbandry and Veterinary Medicine, Beijing Academy of Agriculture and Forestry Sciences, Beijing, China; ^3^ Key Laboratory of Veterinary Bioproduction and Chemical Medicine of the Ministry of Agriculture, Zhongmu Institutes of China Animal Husbandry Industry Co., Ltd., Beijing, China; ^4^ National Institute for Viral Disease Control and Prevention, Collaboration Innovation Center for Diagnosis and Treatment of Infectious Diseases, Chinese Center for Disease Control and Prevention, Key Laboratory for Medical Virology, National Health and Family Planning Commission, Beijing, China

**Keywords:** H1N1 influenza A virus, mast cells, defective viral particles, immunogenicity, protective immune reaction

## Abstract

Mast cells play pivotal roles in the pathogenesis of influenza A virus (IAV) infections. Defective viral particles (DPs) often arise during IAV replication, which can interfere with the replication of infectious viruses and stimulate the antiviral response of host cells. Therefore, DPs are expected to have immune-protective functions in clinic. However, the potent immunogenicity and effectiveness of DPs arising in mast cells during IAV replication have not been reported. In the present study, we showed that DPs generated in the human mastocytoma cell line HMC-1 following H1N1 infection were safe to mice after vaccination. Compared with lung adenocarcinoma cells, A549, DPs generated in infected mast cells had much better immunostimulatory activity, enhancing both humoral and cellular immunity of hosts. Notably, they could significantly increase the expression of immune-associated cytokines, especially the IFN-γ. Due to the robust immunogenicity, thus DPs generated in infected mast cells could stimulate the robust protective immune reaction effectively to fight against lethal IAV re-challenge after vaccination, which result in the high survival, decreased lung injury as well as inhibition of viral replication and inflammatory response in lungs. This study is the first to illustrate and explore the safety, immunogenicity, and effectiveness of DPs arising in mast cells against influenza as favorable potential vaccination. The results provide insight into the advances of new prophylactic strategies to fight inﬂuenza by focusing on DPs generated in mast cells.

## Introduction

Influenza A virus (IAV) is a segmented negative-stranded RNA virus that can infect animals and humans. It consists of eight gene segments (PB2, PB1, PA, HA, NP, HA, M, and NS) that together encode 17 different viral proteins (1, 2). IAV is classified according to two crucial surface glycoproteins encoded by HA and NA (3). H1N1 human influenza virus can cause major epidemics in humans, which raises the perceived public health significance of influenza to a new level. Vaccination remains the most effective preventive measure against influenza viruses. Nevertheless, vaccination effectiveness may be variable from one season to another and seasonal vaccines are not effective against pandemic viruses, and new variants arise through antigenic drifts or shifts (4). Therefore, development of novel broad-spectrum prophylactic agents against IAV is becoming research hotspots in public health and medicine.

Mast cells play an important role in both the innate and adaptive immune response. They are highly granulated cells widely distributed in connective tissue and over the mucosal surface of the body. Mast cells are pivotal not only in immunoglobulin E (IgE)-dependent anaphylaxis, but also in the host’s defense against parasites and bacteria (5, 6). In addition, mast cells have important roles in certain viral infections, such as bovine respiratory syncytial virus (RSV), Newcastle disease virus, and dengue virus (7–9). During dengue virus infection, mast cells can be activated, release various cytokines, induce apoptosis, and then participate in the injury process (10). We previously demonstrate that mast cells are activated in IAV-infected mice and escalated lung injury (11). Besides, our findings highlight the remarkable tropism and infectivity of IAV to P815 cells, indicating that mast cells may be unneglectable player in the development of IAV infection (6).

Defective viral genomes (DVGs) are truncated viral genomes that are produced at the peak of full-length virus replication in various cases such as high multiplicity of infection (MOI) and prolonged infection time (12–14). Von Magnus was the first to identify that influenza virus contained DVGs are also called defective particles (DPs) (15). Compared with infectious virus particles that contain all eight of the full-length gene segments, DPs lose infectivity due to at least one truncated gene segment (16). DVGs mostly occur in PB2, PB1, and PA gene fragments which encode polymerase and M gene segments during IAV infection (17). Recently, a research shows that the HA gene segment is truncated in the H1N1 influenza strain (18). Numerous studies have shown that DVGs can be found in various IAV-infected cells, such as Madin-Darby canine kidney (MDCK) epithelial cells and the human lung adenocarcinoma cell line A549 (18, 19).

DPs have two main functions: interfering with the replication of infectious viruses and stimulating the immune response of host cells, which are considered as potential antiviral agents. The interfering activity of DPs has been reported for various viruses, such as Sendai virus (SeV), RSV, vesicular stomatitis virus (VSV), and influenza virus (13, 16, 20). In addition, DPs strongly induce interferon (IFN) expression in cells during the infection and trigger the antiviral responses to fight viral invasion (21–24). The interfering and IFN-inducing activity of DPs prompt investigating the potential of DVGs as vaccine adjuvants and broad-spectrum antivirals (20, 21, 25). Several reports demonstrate that the mice infected with influenza virus at high DVGs (HD virus) show much lessened pathological sequalae and diminished viral load (26). It can also stimulate a local or systemic immune response in the host, inducing the maturation of dendritic cells and the differentiation of T cells. In addition, DPs that carry the 244 395nt defective segment 1 RNA are found to have an immune-protective effect against influenza B virus infection, which are prepared from plasmids encoding 244 RNA and infectious A/PR/8/34 virus by transfection of 293T cells and co-cultivation with MDCK cells (27–29). These findings confirm that the presence of DPs can limit the course of infection and reduce the excessive inflammatory response, which is worth of further study for extensive application.

In the present study, we isolated and prepared DPs from human mastocytoma cell line, HMC-1 following H1N1 infection, then assessed the immune-protective function of DPs by investigating and analyzing the safety, immunogenicity, and protective effectiveness of these DPs arising in mast cells against influenza after vaccination. Compared with lung adenocarcinoma cells, A549, DPs generated in H1N1-infected HMC-1 cells were safe and could trigger a robust adaptive immunity and enhance the immune system of mice protected from lethal H1N1. This is the first study to reveal the pivotal role of DPs generated in mast cells on protecting mice from IAV infection as favorable potential vaccination. The results provide insight into the advances of new prophylactic strategies to fight inﬂuenza by focusing on DPs generated in mast cells.

## Materials and Methods

### Viruses and Cell Culture

The H1N1 (A/WSN/33) virus was isolated and virus titers were determined as described previously (30). The stocks used for originally infection had been purified with low content of DVGs (LD virus). The HMC-1 cells were provided by Medical College of China Three Gorges University. The A549 cells and the MDCK cells were provided by the Cell Resource Center of Peking Union Medical College (Beijing, China) and cultured as described previously (31). For LD virus preparation, the virus was propagated in MDCK cells at 37°C for 48 h, and the viral supernatant was harvested, aliquoted, and stored at −80°C. The infectivity titer of the supernatant was measured in MDCK cells following serial dilution of the stock using half-maximal tissue culture infectious dose (TCID50) assays and total titer was measured using hemagglutination assays.

### Virus With High Content of DVGs (HD Virus) Isolation and Immunization

The HMC-1 cells and A549 cells were infected with H1N1 at an equal MOI of 10 for 30 and 18 h, respectively, and then the cell supernatants were collected, purified by differential centrifugation (3,000r 30min, 10,000r 30min) and DPs were detected by titer infectivity (I)/total titer (T) ratio calculation as described previously (18). Generally, the quantitative measure of the DPs can be achieved by calculating the ratio which is equal to infectious particles/total viral particles. The infectivity titers of the culture media supernatants were measured using TCID50 assays and total titers were measured using hemagglutination assays. The I/T ratios equaled to TCID50 per 25 µl/HA per 25 µl. These samples were called the HD virus that contained high content of DVGs.

For immunization, mice were anesthetized with Zotile^®^ (Virbac, France) and intranasally infected with 10 TCID50/mouse H1N1 HD virus. The control mice were treated with PBS or Dulbecco’s modified Eagle’s medium (DMEM) alone. The serum and spleens were collected on day 14 and 21 (three mice per group each time) after immunization and stored at −80°C.

### Detection of Serum Immunoglobulins

The double-antibody sandwich enzyme-linked immunosorbent assay (ELISA) was used to determine the serum titers of total IgG, IgG1, and IgG2a in accordance with the instructions of kits. The catalog numbers of Mouse IgG ELISA Kit, Mouse IgG1 ELISA Kit, and Mouse IgG2a ELISA Kit were 70-EK2712, 70-EK2722, and 70-EK2732, which were purchased from Multi Sciences Company in China. Briefly, plates were precoated with the capture antibody specific for IgG, IgG1, and IgG2a generated by immunization of mouse, respectively. Next, the serum solution is incubated with the capture antibody to facilitate binding. The plates were washed and added with substrate TMB. Then, stop solution was added to each well of the plates. The absorbance was determined at 450 nm.

### Hemagglutination Inhibition (HI) Test

HI test was carried out to detect influenza virus antibodies in serum samples as previously described (32). Briefly, four hemagglutinating units (HAU) of virus were prepared according to the HA titers, then serum samples with a two-fold dilution were mixed 1:1 with 25 µl 4 HAU virus, the mixture was incubated at room temperature for 15 min, then 50 µl erythrocytes were added and incubated at room temperature for the appropriate time. The HI titer was the reciprocal of the last dilution of the serum that completely inhibits hemagglutination.

### Fluorescence-Activated Cell Sorting (FACS) Analysis of T Cell Cytokine Production

Mice were sacrificed and single cell suspensions were isolated from the spleens. Then, FACS analysis was performed to determine T cell cytokine production, as described previously (33). The following antibodies were ordered from eBiosciences (San Diego, CA, USA): CY5-conjugated anti-CD4, CY5-conjugated anti-CD8, FITC-conjugated anti-IFN-γ, and FITC-conjugated anti-IL-4.

### Real-Time Quantitative PCR (RT-qPCR)

The expression of viral HA genes, IFN-β, IFN-γ, ISG56, monocyte chemotactic protein 1 (MCP-1), tumor necrosis factor (TNF), and interleukin 6 (IL-6) was determined as described previously (33). The primer sequences were listed in **Table S1**.

### The Detection of T-Cell Proliferation

Single lymphocyte suspensions were isolated from the spleens of mice in each group (n = 3) at day 21 after the immunization. The methyl thiazolyl tetrazolium (MTT) method and MTS method were used to measure T-cell proliferation as previously described (32, 34). For MTT method, single-lymphocyte suspensions were seeded into 96-well plates (1 × 10^6^ cells/well) in 100 μl of medium for indicated time. Then, 10 μl MTT was added to each well, and cells were incubated for 4 h at 37°C. Supernatant was removed, and 100 μl DMSO was added to dissolve the formazan production. Well absorbance was read on a microplate reader. For MTS method, single-lymphocyte suspensions were incubated in triplicate in 96-well plates at 5 × 10^4^ cells/well in RPMI-1640 containing 5% of fetal calf serum at 37℃ in a 5% CO2 incubator and stimulated for 48 h with 2 µg/ml BSA. Cells that were not added with BSA were regarded as a negative control. Following stimulation, a 20 µl mixture of (3-(4,5-dimethylthiazol-2-yl)-5-(3-carboxymethoxyphenyl) -2-(4-sulfophenyl) -2H-tetrazolium inner salt)/phenazine methosulfate (MTS/PMS) reagent was added and incubated for 4 h, then the optical density values were read by a plate reader. Data were expressed as the stimulation index, calculated as the mean reading of triplicate wells of antigen stimulation divided by the mean reading of triplicate wells of the negative control.

### Viral Challenge In Vivo

Female BALB/c mice aged 6–8 weeks were anesthetized with Zotile^®^ and intranasally infected with 120 TCID50/mouse H1N1 LD virus at day 21 after the immunization. The mice were observed for 14 days after the viral challenge. Three mice in each group were sacrificed at day 3 and day 6 post infection, respectively. The lung tissues were collected and some were stored at −20°C.

### Ethics Statement

Animal experiments were approved by the Animal Ethics Committee of China Agricultural University (approval number 201206078) and were performed in accordance to Regulations of Experimental Animals of Beijing Authority. Besides, experimental protocols conformed to the guidelines of the Beijing Laboratory Animal Welfare and Ethics Committee and were approved by the Beijing Association for Science and Technology (approval number SYXK-2009-0423).

### Histopathological and Immunohistochemical Analysis

Tissues were fixed with 4% neutral formalin for 48 h at room temperature, embedded in paraffin, and then cut to 5 μm thickness. The detection of histopathological changes by hematoxylin and eosin (H&E) staining and immunohistochemical (IHC) staining were reported previously (33). Anti-IAV nucleoprotein (NP) mAb (Abcam) were used in IHC staining and were diluted at 1:1,000.

Pathological changes were evaluated by a veterinary pathologist and scored from 0 to 4 in a blind study. Descriptions of the scores were as follows: 0 = no microscopic lesions; 1 = extremely mild, characterized by slight hyperemia and hemorrhage; 2 = mild, characterized by hemorrhage, hyperemia, and desquamation of rare epithelial cells; 3 = moderate, characterized by hemorrhage, hyperemia, desquamation of many epithelial cells, and slight infiltration of inflammatory cells; 4 = severe, characterized by hemorrhage, hyperemia, desquamation of many epithelial cells, and greater infiltration of inflammatory cells. Detection of the IAV antigen was scored from 0 to 4 according to the number of positive cells per section. Descriptions of the scores were as follows: 0 = no positive cells; 1 = 1–10 positive cells; 2 = 11–30 positive cells; 3 = 31–50 positive cells; 4 = >50 positive cells.

### Plaque Assays

The procedures of plaque assays were the same as previous described. The plaques were counted and the concentration of the initial viral suspension in plaque forming units (PFU)/ml was calculated.

### Data Analysis

Data analysis was conducted using two-way analysis of variance (ANOVA) with GraphPad Prism 5.0. P < 0.05 represents statistical significance. The results were showed as the means ± standard deviations of three independent experiments.

## Results

### The Safety of HD Virus Immunization In Vivo

**Figure 1:**
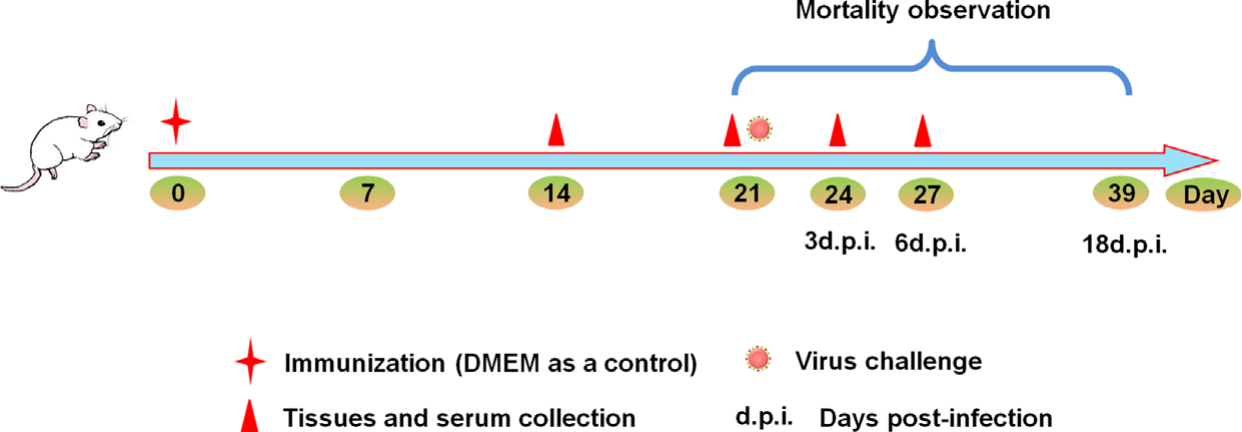
Experimental design schematic. Mice were randomly divided into four groups. Mice were immunized with 10TCID50/mouse HD virus (HMC-1 cells), HD virus (A549 cells), LD virus (or DMEM) at day 0, and all the mice were challenged with 120TCID50/mouse of LD virus on day 21. The body weight and mortality of mice were observed during the whole process. Three mice from each group were sacrificed on day 14, 21, 24, and 27, serum, spleen and lung tissues were collected.

**Figure 2:**
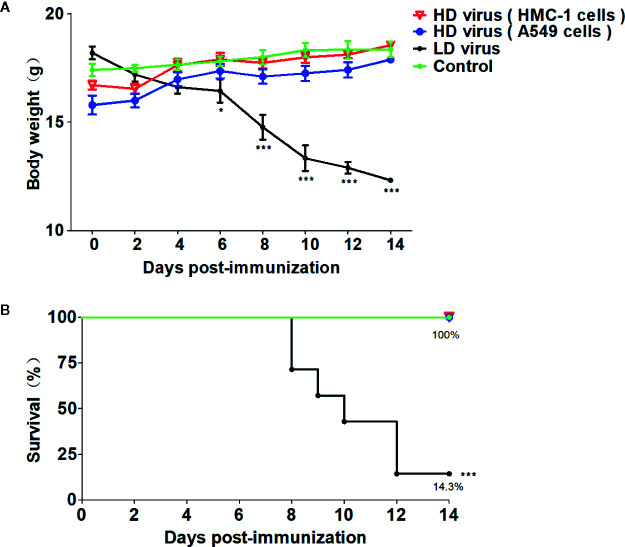
The safety of HD virus immunization *in vivo*. **(A)** Weight loss and **(B)** survival rates of mice were recorded during HD virus immunization (N = 7). Statistical difference was analyzed by comparing each group with control group. (*P < 0.05; ***P < 0.001).

Firstly, HD viruses that contained high content of DVGs were isolated and prepared from HMC-1 cells and A549 cells following H1N1 infection, respectively. As shown in **Table S2**, H1N1 virus stocks that maintained a low DVGs content was regarded as LD virus. HD viruses isolated from H1N1-infected HMC-1 cells and A549 cells had the same I/T ratios, which were much lower than found in LD virus. Then, the safety and immune-protective effects of prepared HD viruses were performed *in vivo*. As outlined in **Figure 1**, mice were divided into four groups and were immunized with 10TCID50/mouse HD virus (HMC-1 cells), HD virus (A549 cells), or treated with LD virus, or DMEM as a control. At 21 days after the vaccination, mice were re-challenged with a lethal dose (120TCID50/mouse) of LD virus. In order to evaluate the safety of HD virus immunization *in vivo*, the clinical manifestations, body weight, and mortality were observed and recorded for 14 days after the vaccination (**Figure 2**). As of 14 days post-immunization, mice in the both HD virus (HMC-1 cells) group and HD virus (A549 cells) group as well as control group were active and displayed a glossy coat, which have a normal weight and 100% survival. In contrast, mice in the LD virus group displayed poor condition such as thinness, depressed activity levels, and ruffled hair, which showed the dramatically dropped body weight and high mortality at 85.7%. Here, we also detected the safety of mice treated with different doses of LD virus (10, 5, 1, 0.5, 0.1, 0.05, 0.01TCID50/mouse) and found that only LD virus at dose of 0.05TCID50/mouse or lower could not cause mice death (**Table S3**). Thus, the results demonstrate that these two HD viruses are safe and have no negative effect on mice during immunization and could be applied in the following experiments.

### DPs Generated in H1N1-Infected Mast Cells Promote the Development of Humoral Immunity

**Figure 3:**
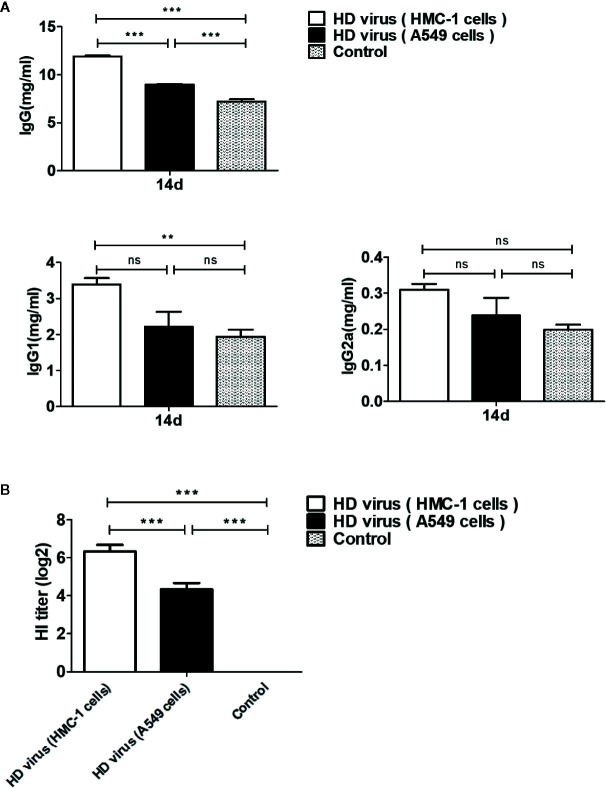
DPs generated in H1N1-infected mast cells promote the development of humoral immunity. Serum samples of mice were collected at 14 days after immunization. **(A)** The IgG, IgG1, and IgG2a titers were conducted by ELISAs. **(B)** Antibody levels against the H1 subtype antigen were taken by HI tests. Graphs are shown from three independent replicates (**P < 0.01; ***P < 0.001; ns, no significance).

To investigate whether DPs generated in H1N1-infected mast cells could influence the immune function of mice after immunization, ELISAs were conducted to detect the levels of immunoglobulins in serum samples. As shown in **Figure 3A**, IgG, IgG1, and IgG2α titers in the HD virus (HMC-1 cells) group increased significantly at 14 d after immunization, and the titers were higher than those in the HD virus (A549 cells) group, especially IgG (P < 0.001). Furthermore, HI tests were also taken to measure the levels of H1 subtype antibodies in serum. As shown in **Figure 3B**, there was a strong HI antibody response to H1N1 in sera taken at 14 days after immunization with HD viruses. The titers of HD virus (HMC-1 cells) group were significantly higher than that of HD virus (HMC-1 cells) group (P < 0.001). Thus, these data suggest that DPs generated in H1N1-infected HMC-1 cells can positively affect virus-related antibody production and promote the antibody response to the H1N1 hemagglutinin in the host.

### DPs Generated in H1N1-Infected Mast Cells Trigger a Robust Cellular Immunity

**Figure 4:**
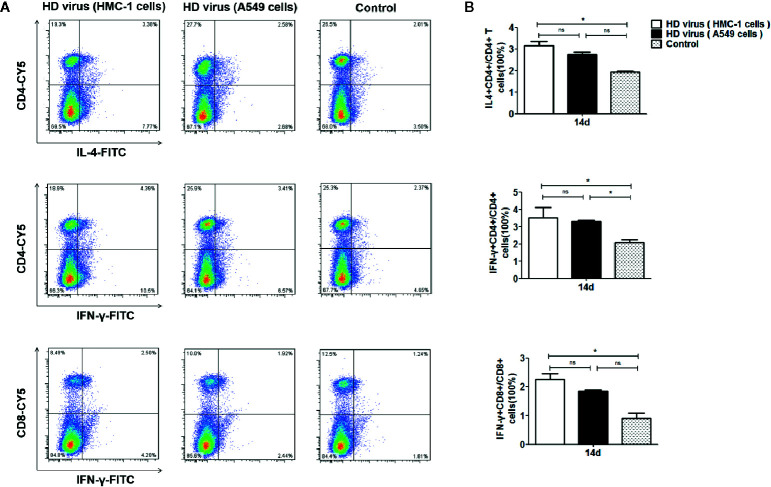
DPs generated in H1N1-infected mast cells promote the T cell response. **(A, B)** T cells from the spleens were collected at 14 days after immunization, stained with different fluorescent antibodies, and analyzed by flow cytometry. Graphs are shown from three independent replicates (*P < 0.05; ns, no significance).

**Figure 5:**
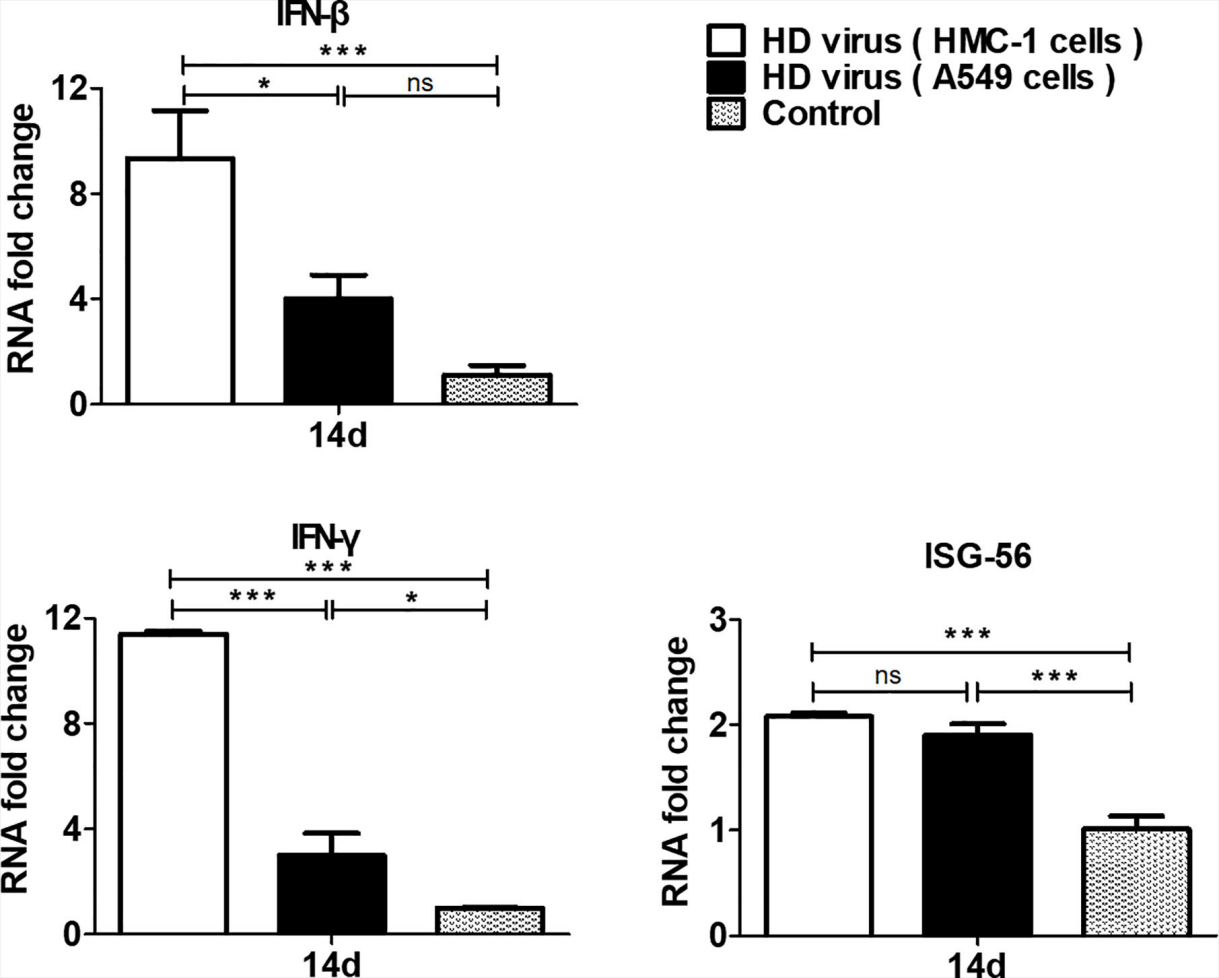
DPs generated in H1N1-infected mast cells upregulate the production of antiviral cytokines. The spleens were collected at 14 days after immunization and the expressions of IFN-β, IFN-γ, and ISG56 were analyzed by RT-qPCR. Graphs are shown from three independent replicates (*P < 0.05; ***P < 0.001; ns, no significance).

To measure the role of DPs on T cell responses, we isolated the single cell suspensions from the spleens of mice after immunization. The effect of DPs on T cell cytokine production was also examined by flow cytometry (**Figure 4**). The expression of antigen-induced IFN-γ and IL-4 in CD4+ T cells was significantly promoted in the HD virus (HMC-1 cells) group (P < 0.05). These results suggest that HD virus (HMC-1 cells) can promote the production of both Th1 and Th2 cytokines. Besides, a higher level of CD8+ T cells that express intracellular IFN-γ was observed in mice in the HD virus (HMC-1 cells) group (P < 0.05), indicating that DPs generated in H1N1-infected HMC-1 cells may facilitate the antigen-specific cytotoxic T lymphocytes (CTLs) response. Moreover, the antiviral response was also assessed by measuring the mRNA expression profiles of IFN-β, IFN-γ, and ISG56 using RT-qPCR (**Figure 5**). The production of antiviral cytokines was upregulated notably in the HD virus (HMC-1 cells) group compared with the any other groups, especially for IFN-γ (P < 0.01), which were in accordance with the results of flow cytometry described above.

**Figure 6:**
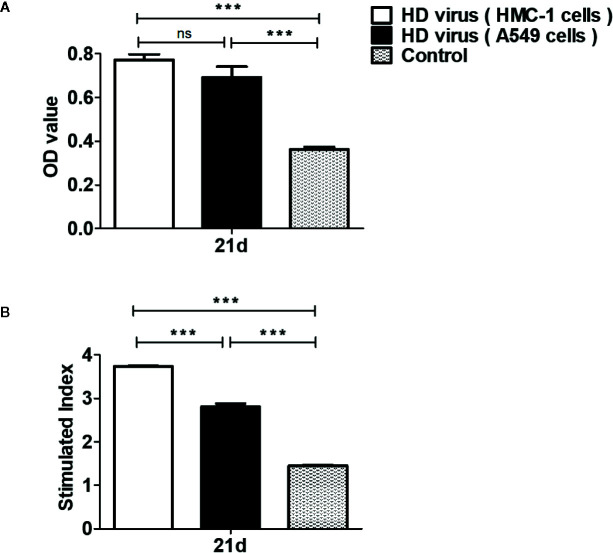
DPs generated in H1N1-infected mast cells promote the T cell proliferative response. T cells from the spleens were collected at 14 days after immunization. **(A, B)** The T cell proliferation was measured using MTT method and MTS method, respectively. Graphs are shown from three independent replicates (***P < 0.001; ns, no significance).

Furthermore, T cell proliferative response was detected to deeply investigate the effects of DPs on cellular immunity. MTT results showed that the OD values in two HD virus groups were all significantly higher than that in control group (P < 0.01) (**Figure 6A**). Though HD virus (HMC-1 cells) group had higher values than HD virus (A549 cells) group, no significance could be found in these two groups. At the same time, MTS method was also applied to measure the T cell proliferative response (**Figure 6B**). The stimulated index in HD virus (HMC-1 cells) group was dramatically promoted compared with any other two groups, indicating that the T cell proliferative response was robustly enhanced in the HD virus (HMC-1 cells) group (P < 0.001). Collectively, these results support that DPs generated in H1N1-infected HMC-1 cells can positively affect the host cell-mediated immunity induced by vaccination, which triggers a robust cellular immunity.

### DPs Generated in H1N1-Infected Mast Cells Have a Strong Protective Efficacy on Mice to Fight Against IAV

**Figure 7:**
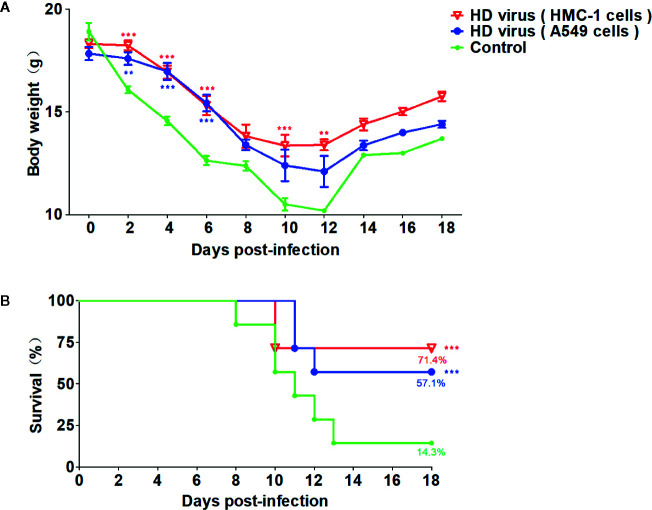
Virus preparations with high number of DPs generated in H1N1-infected mast cells have a strong protective efficacy on mice to fight against IAV. Mice were re-challenged with 120TCID50/mouse H1N1 LD virus at 21 days after the first vaccination. **(A)** Weight loss and **(B)** survival rates of infected mice were recorded (N = 7). Statistical difference was analyzed by comparing each group with control group (**P < 0.01; ***P < 0.001).

On the basis of the robust humoral and cellular immunity of DPs generated in H1N1-infected HMC-1 cells, thus we detected whether vaccination of these DPs could provide a protection against the serious disease caused by lethal IAV infection. As mentioned above, mice were re-challenged with 120TCID50/mouse H1N1 LD virus at 21 days after the first vaccination, then the body weight and mortality were assessed for 14 days. As shown in **Figure 7**, after re-challenge, mice that received HD virus (HMC-1 cells) had milder clinical manifestations, slighter weight loss, and higher survival than any other group. The survival rate of the HD virus (HMC-1 cells) group was 71.4% and was lower than those of the HD virus (A549 cells) (57.1%) and control group (14.3%). Besides, we also detected the protective efficacy of LD virus at dose of 0.05 and 0.01TCID50/mouse, and found that LD virus at either of these safe doses have no protective efficacy on mice to fight against IAV (**Table S3**). Above all, the results suggest that DPs vaccination strategy was protective.

**Figure 8:**
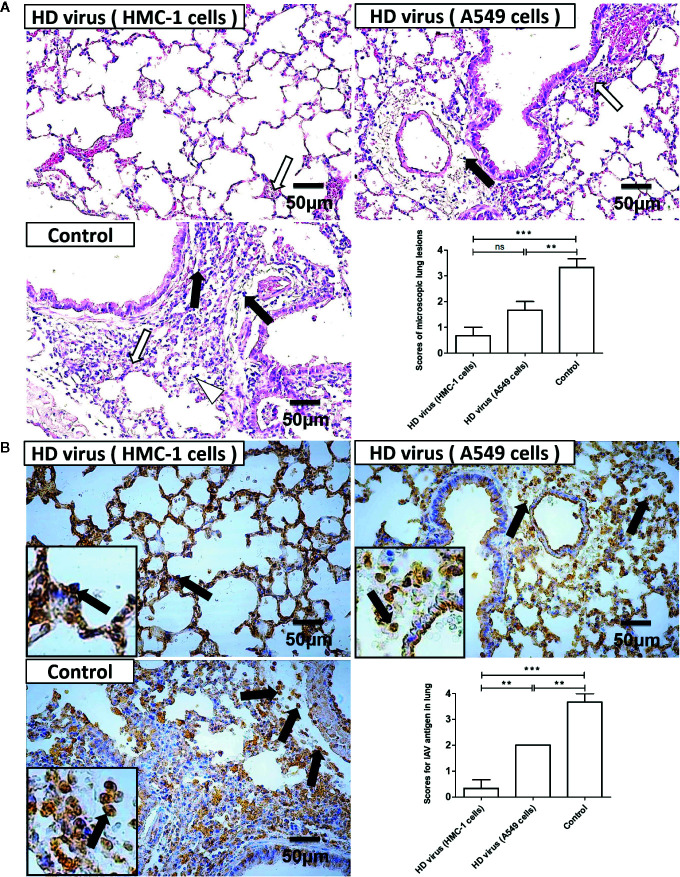
The immunization of virus preparations with high number of DPs generated in H1N1-infected mast cells could decrease the lung injury and viral load in infected mice. The lungs were collected at day 6 post infection. **(A)** Lung pathological changes were observed by H&E staining and scored in a blind study. Black arrows indicate lymphocytic infiltration. Hollow arrows indicate hemorrhage and hyperemia. Hollow triangles indicate desquamation of epithelial cells. **(B)** The expression of viral NP was measured using IHC staining and scored in a blind study. Black arrows indicate positive signals. Graphs are shown from three independent replicates (**P < 0.01; ***P < 0.001; ns, no significance).

Besides, histopathological changes in the lungs were also examined by H&E staining (**Figure 8A**). As expected, the lungs of mice in the control group had severe lesions, including hyperemia, hemorrhage, and desquamation of epithelial cells as well as many lymphocytes and inflammatory cells infiltration surrounded the bronchioles and blood vessels. Compared with the control group, HD virus (A549 cells) group showed the milder histopathological changes, such as fewer lymphocytes and inflammatory cells infiltration. No obvious lesions were seen in the HD virus (HMC-1 cells) group, and only slight hemorrhage was present in the alveoli. The scores of pathological changes in lungs of mice were also determined. As shown in **Figure 8B**, the IHC staining results by using an IAV NP antibody also showed fewer positive cells in lung of mice in the HD virus (HMC-1 cells) group and HD virus (A549 cells) group when compared to the control group, especially that in the HD virus (HMC-1 cells) group. The scores of IAV antigens in the lungs of H5N1-infected mice were determined and could further show the significant difference between these two HD virus groups. The results suggested that DPs generated in H1N1-infected HMC-1 cells can alleviate lung injury in infected mice.

**Figure 9:**
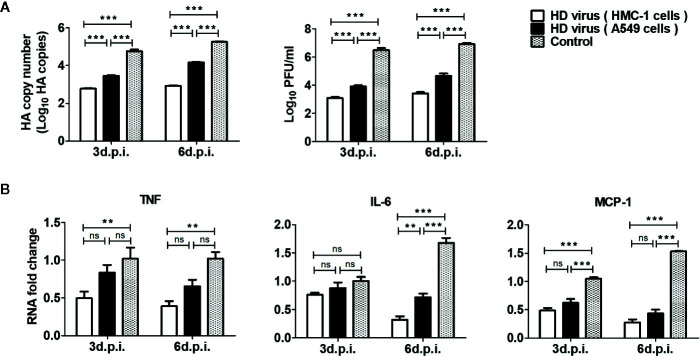
The immunization of virus preparations with high number of DPs generated in H1N1-infected mast cells could inhibit the viral replication and inflammatory response in infected mice. The lungs were collected at day 3 and day 6 post infection. **(A)** The HA copy numbers and viral titers were measured by RT-qPCR and plaque assay, respectively. **(B)** The expressions of IL-6, TNF, and MCP-1 were analyzed by RT-qPCR. Graphs are shown from three independent replicates (**P < 0.01; ***P < 0.001; ns, no significance).

To investigate the influence of vaccination on replication and viral loads of IAV *in vivo*, the lung tissues of mice at day 3 and day 6 post-infection were collected and examined by RT-qPCR and plaque assay. As shown in **Figure 9A**, HA copy numbers and PFU values in the HD virus (HMC-1 cells) group were significantly lower than those in the HD virus (A549 cells) or control group (P < 0.001). These viral titer results were in accordance with the results of IHC staining and further demonstrated that DPs generated in H1N1-infected HMC-1 cells can reduce viral replication and viral loads in infected mice.

As excessive levels of pro-inflammatory cytokines induce an acute mononuclear and neutrophilic inflammatory response, therefore inflammatory cytokines in the lungs of mice were determined by RT-qPCR. As shown in **Figure 9B**, the HD virus (HMC-1 cells) group showed the lowest expressions of inflammatory cytokines among these groups, indicating the reduced inflammatory response. In terms of IL-6, the significant differences could be found between HD virus (HMC-1 cells) group and HD virus (A549 cells) (P < 0.01) at day 6 post-infection. Collectively, these results support that DPs generated in H1N1-infected HMC-1 cells can have a strong protective efficacy on mice to fight against IAV through decreased lung injury as well as inhibition of viral replication and inflammatory response.

## Discussion

Mast cells, one kind of critical immune cells, are known to have pivotal roles in both innate and protective immunity against viral infections *via* releasing various cytokines and chemokines (5, 35, 36). DPs can be generated in various kinds of cells and have two crucial functions: interfering with full-length virus replication and inducing antiviral responses in various cells during IAV infection, which are considered as potent vaccine adjuvants and broad-spectrum antivirals (20, 25). Previous studies have shown that DPs originated from the mouse Sendai virus can be regarded as potent immunostimulants in the process of immunization (16). These findings confirm that the presence of DPs can limit the course of infection and reduce the inflammatory response. Here, we also detected the immunostimulatory properties of IAV DPs. The object of our research was evaluating the immunogenicity and effectiveness of DPs generated in mast cells against influenza after vaccination, to further investigate whether DPs generated in H1N1-infected mast cells also have the potential immunostimulatory. Under conditions with the same content of viral titers and DPs, the vaccination of DPs generated in H1N1-infected HMC-1 cells were safe to mice, which had no negative effects such as body weight and mortality.

It is well known that the protective immunity against viral infections is correlated with antigen-specific humoral responses (37). Here, we investigated the effects of DPs generated in H1N1-infected HMC-1 cells on humoral response. We found that total IgG, IgG1, and IgG2a as well as HI serum titers were all increased at day 14 after immunization in HD virus (HMC-1 cells) group compared with those in HD virus (A549 cells). Therefore, our study is the first to confirm that DPs generated in H1N1-infected HMC-1 cells can promote the antibody production in serum and enhance the antigen-specific humoral responses in the host.

Furthermore, the protective immunity against viral infections is associated with antigen-specific cellular responses, including the expression of T cell related cytokines and the T cell proliferation (37, 38). IFN-γ, a type II IFN, has a pivotal role in regulating immunity. During viral infection, both antigen-specific humoral and cell-mediated immunity are triggered in the hosts. Mature T cells are classified into two subgroups: CD4+ T cells that recognize the exogenous antigen, and CD8+ T cells that recognize the endogenous antigen. IFN-γ is produced and secreted from both types of T cell but performs different functions in each. IFN-γ secreted from CD4+ T cells promotes the activation of T cells, while IFN-γ secreted from CD8+ T cells enhances the ability of cytotoxic T cells to kill abnormal cells. Our previous study shows that IFN-γ can also be released from mouse mast cells during IAV infection and regulate a series of pulmonary epithelial cell apoptosis (6). To date, various reports have shown that DPs can stimulate the immune response *via* high-level expression of type I IFN (39, 40). However, few reports have assessed the action of type II IFN in the immune response induced by DPs. Considering the crucial role of IFN-γ in the cellular response, we also detected not only antigen-induced IL-4 but also IFN-γ in CD4+ T cells and CD8+ T cells, respectively. The results showed that DPs generated in H1N1-infected HMC-1 cells could trigger a robust cellular response, especially the antigen-specific CTLs response. RT-qPCR results could further confirm the significant higher expression of IFN-γ in HD virus (HMC-1 cells) group compared with those in HD virus (A549 cells) at day 14 after immunization. Here, we provide novel data demonstrating that DPs generated in H1N1-infected HMC-1 cells could trigger a robust cellular response. In addition, T cell proliferative response was robustly enhanced in the HD virus (HMC-1 cells) group. Together, these results firstly validate that DPs generated in H1N1-infected HMC-1 cells can trigger a robust cellular immunity, including the promotion of T cell proliferation and related cytokines, especially the IFN-γ.

To further understand if the enhanced levels of the humoral and cellular responses induced by DPs generated in H1N1-infected HMC-1 cells could have the protective efficacy on mice to fight against IAV, mice were re-challenged with lethal H1N1 LD virus at 21 days after the first vaccination. After re-challenge, DPs generated in H1N1-infected HMC-1 cells could make the slighter weight loss and higher survival when compared with DPs generated in infected A549 cells. In lung lesions, decreased lung injury as well as inhibition of viral replication and inflammatory response was induced by DPs generated in H1N1-infected HMC-1 cells. Thus, DPs generated in H1N1-infected HMC-1 cells have a strong protective efficacy on mice to fight against IAV. *Nigel J. Dimmock* et al. demonstrates that cloned DPs, which carry the 244 395nt defective segment 1 RNA and are amplified in MDCK cells, can not only protect mice from lethal *in vivo* infection with IAVs from several different subtypes, but also protect *in vivo* against genetically unrelated interferon-sensitive respiratory viruses such as pneumonia virus of mice through inducing a protective immune response (21, 41). Thus, we speculate that these robust immune-protective effects induced by DPs generated in H1N1-infected HMC-1 cells can also have the potential to protect against various subtypes of IAVs as well as respiratory virus infections. Notably, LD virus at either of these safe doses had no protective efficacy on mice to fight against IAV, further indicating that it is the defective virus particles rather than a small portion of the infectious viruses in the HD virus samples that induced the great protection. Besides, we speculate that different immune-protection of DPs generated in H1N1-infected HMC-1 cells and A549 cells mainly because of the differences of DVGs in DPs generated in these two different kinds of cells. We consider that DPs generated in H1N1-infected HMC-1 cells contain one or some unique deleted RNA species that could not be found in DPs generated in infected A549 cells.

**Figure 10:**
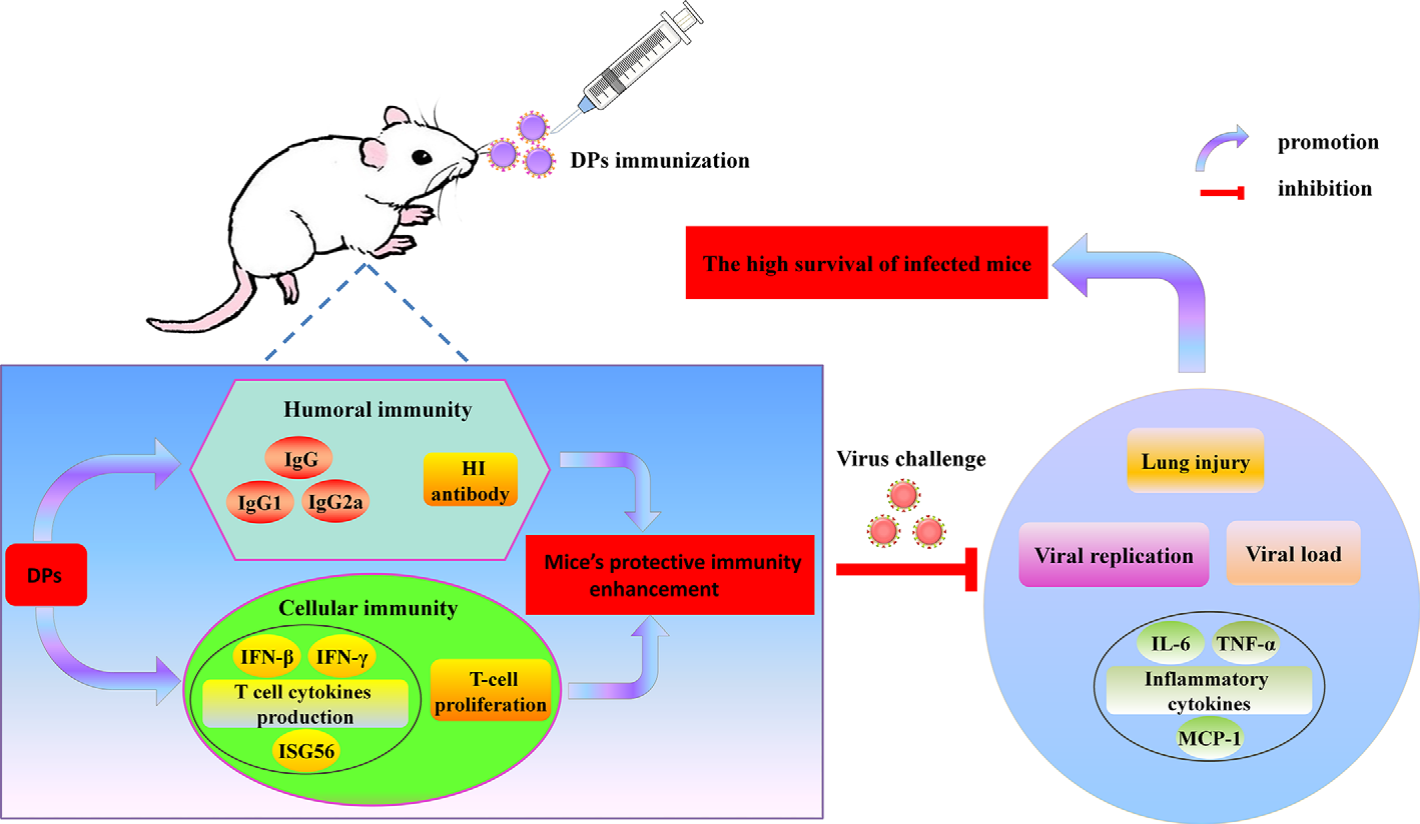
Overview of findings.

In summary, as shown in **Figure 10**, our data suggests that DPs generated in DPs generated in H1N1-infected mast cells facilitate to trigger adaptive immune responses of mice and have much better immunostimulatory activity than those generated in infected A549 cells, enhancing both humoral and cellular immunity of hosts and stimulating the robust protective immune reaction against lethal IAV re-challenge. The protection afforded by these DPs has clinical beneﬁts against diseases caused by IAV infection. The advantages of DPs as antivirals include their efﬁcacy, single dose requirement, low amounts of material, immediate effect, and protection. These observations demonstrate for the first time that DPs generated in H1N1-infected mast cells are potent novel candidate immunostimulants that should be tested in various vaccine platforms and can be beneﬁcial in the prophylaxis of IAV infection.

## Data Availability Statement

The original contributions presented in the study are included in the article/**Supplementary Material**. Further inquiries can be directed to the corresponding authors.

## Ethics Statement

The animal study was reviewed and approved by Beijing Laboratory Animal Welfare and Ethics Committee, Beijing Association for Science and Technology.

## Author Contributions

CH, JT, JC, and YH carried out experiments, analyzed data, and wrote the paper. JX, MC, SZ, HT, MW, HS, and YH designed the study and supervised the project. CH, HS, and YH assisted in the data analysis and discussion. CH and YH drew the figures. All authors contributed to the article and approved the submitted version.

## Funding

Research reported in this publication was supported by the National Natural Science Foundation of China (Grant no. 31772702) and the Chinese Universities Scientific Fund (Grant no. 2018TC044) as well as the Talent Cultivation Program of China Agricultural University.

## Conflict of Interest

Author JX was employed by the company Zhongmu Institutes of China Animal Husbandry Industry Co., Ltd.

The remaining authors declare that the research was conducted in the absence of any commercial or financial relationships that could be construed as a potential conflict of interest.
