# Supplementary material for: Safety, Immunogenicity, and Effectiveness of Defective Viral Particles Arising in Mast Cells Against Influenza in Mice
Source: Front Immunol. 2020 Nov 13;11:585254. doi: 10.3389/fimmu.2020.585254 (PMC7693459; doi:10.3389/fimmu.2020.585254)
Supplement: Supplementary file 1 [file Table_1.pdf]

Supporting information

Safety, immunogenicity and effectiveness of defective viral particles arising in mast cells against influenza in mice

Caiyun Huo[1,2†], Jijing Tian[1†], Jinlong Cheng[1], Jin Xiao[3], Mingyong Chen[1], Shumei Zou[4], Haiyan Tian[1], Ming Wang[1], Huiling Sun[2*], Yanxin Hu[1*]

[1] Key Laboratory of Animal Epidemiology of Ministry of Agriculture, College of Veterinary Medicine, China Agricultural University, Beijing, China.

[2] Beijing Key Laboratory for Prevention and Control of Infectious Diseases in Livestock and Poultry, Institute of Animal Husbandry and Veterinary Medicine, Beijing Academy of Agriculture and Forestry Sciences, Beijing, China.

[3] Key Laboratory of Veterinary Bioproduction and Chemical Medicine of the Ministry of Agriculture, Zhongmu Institutes of China Animal Husbandry Industry Co., Ltd, Beijing, China.

[4] National Institute for Viral Disease Control and Prevention, Collaboration Innovation Center for Diagnosis and Treatment of Infectious Diseases, Chinese Center for Disease Control and Prevention, Key Laboratory for Medical Virology, National Health and Family Planning Commission, Beijing, China.

† Contributed to the work equally.

*** Corresponding authors:**

Dr. Yanxin Hu, Key Laboratory of Animal Epidemiology of Ministry of Agriculture, College of Veterinary Medicine, China Agricultural University, No. 2 Yuanmingyuan West Road, Beijing, 100093, China. Email: 07033@cau.edu.cn

Dr. Huiling Sun, Beijing Key Laboratory for Prevention and Control of Infectious Diseases in Livestock and Poultry, Institute of Animal Husbandry and Veterinary Medicine, Beijing Academy of Agriculture and Forestry Science, #9 Shuguang Huayuan Zhonglu, Haidian District, Beijing, 100097, China. Email: sunhuiling01@163.com

Table S1. The primer sequences

	Target name	Primers
**Influenza A virus**	**HA**	**5'- CGCAGTATTCAGAAGAAGCAAGAC -3'**
		**5'- TCCATAAGGATAGACCAGCTACCA -3'**
**Mouse**	**IFN-β**	**5'- AGATGTCCTCAACTGCTCTC -3'**
		**5'- AGATTCACTACCAGTCCCAG -3'**
	**IFN-γ**	**5'- GACTGTGATTGCGGGGTTGT -3'**
		**5'- GGCCCGGAGTGTAGACATCT -3'**
	**ISG56**	**5'- GACAAGGCAATCACCCTCTAC -3'**
		**5'- GTCTTTCAGCCACTTTCTCCA -3'**
	**TNF**	**5'- TCACTGGAGCCTCGAATGTC -3'**
		**5'- GTGAGGAAGGCTGTGCATTG -3'**
	**IL-6**	**5'- ACAGAAGGAGTGGCTAAGGA -3'**
		**5'- CGCACTAGGTTTGCCGAGTA -3'**
	**MCP-1**	**5'- GCTTCTGGGCCTGCTGTTCA -3'**
		**5'- AGCTCTCCAGCCTACTCATT -3'**
	**β-actin**	**5'- GAGACCTTCAACACCCCAGC -3'**
		**5'- ATGTCACGCACGATTTCCC -3'**

Table S2. I/T ratios in HD virus isolated from HMC-1 cells and A549 cells following H1N1 infection.

	Time of sample collection	Infectivity titer (I): TCID50/25μl	Total titer (T): HA/25 μl	I/T
HD virus (HMC-1)	30h	6264.84	16	391.5525
HD virus (A549)	18h	6264.84	16	391.5525
LD virus	/	9929102.93	256	38785.55832

Table S3. The safety of mice treated with different doses of H1N1 LD virus and their protective efficacy on mice to fight against IAV. Mice were firstly treated with different doses of H1N1 LD virus and re-challenged with 120TCID50/mouse H1N1 LD virus at 21 days. The survival rates of infected mice were recorded.

TCID50/	Days post-challenge							Days post-rechallenge (120TCID50/mouse)					
mouse	8	9	10	11	12	13	14	9	10	11	12	13	14
10	5/7	4/7	3/7	3/7	1/7	1/7	1/7	/	/	/	/	/	/
5	6/7	6/7	4/7	3/7	2/7	2/7	2/7	/	/	/	/	/	/
1	7/7	6/7	5/7	5/7	3/7	2/7	2/7	/	/	/	/	/	/
0.5	7/7	7/7	7/7	6/7	5/7	4/7	4/7	/	/	/	/	/	/
0.1	7/7	7/7	7/7	6/7	6/7	6/7	5/7	/	/	/	/	/	/
0.05	7/7	7/7	7/7	7/7	7/7	7/7	7/7	5/7	5/7	3/7	2/7	2/7	2/7
0.01	7/7	7/7	7/7	7/7	7/7	7/7	7/7	6/7	4/7	4/7	3/7	2/7	2/7
