# Supplementary material for: Safety, Immunogenicity, and Effectiveness of Defective Viral Particles Arising in Mast Cells Against Influenza in Mice
Source: Front Immunol. 2020 Nov 13;11:585254. doi: 10.3389/fimmu.2020.585254 (PMC7693459; doi:10.3389/fimmu.2020.585254)
Supplement: Supplementary file 2 [file Table_2.docx]

**Response:**

Dear editor,

Thank you very much for your willingness to giving us this valuable opportunity. Here, we have combined the results of Fig.2 and Fig.7 into a single figure below, which could show the whole experimental process. At day 0, HD virus groups were immunized with 10 TCID50/mouse HD virus, LD virus group was treated with 10 TCID50/mouse LD virus. As a control, the Control group was treated with DMEM. At day 21, all groups were viral challenged with 120 TCID50/mouse LD virus. We could clearly observe the survival of all groups. In order to make the manuscript more accurate, we have modified the “Immunization” to “Immunization (DMEM as a control)” in Fig.1 to make the figure more accurate. The corrections could be seen in new Fig.1.

Additionally, we really apologize for several grammatical and syntax errors. As editor’s suggested, we have corrected the related errors very carefully and the changes were marked in red color in the revised manuscript.

Here, thanks very much for these valuable suggestions again. Thanks for your kind helps.
